# The mediating effect of distress tolerance on the relationship between stressful life events and suicide risk in patients with major depressive disorder

**DOI:** 10.1186/s12888-023-04600-7

**Published:** 2023-02-22

**Authors:** Jing Zhong, Xiao-Jie Huang, Xue-Mei Wang, Ming-Zhi Xu

**Affiliations:** 1 School of Public Health, Southern Medical University, Guangzhou, Guangdong People’s Republic of China; 2 Guangdong Mental Health Center, Guangdong Provincial People’s Hospital (Guangdong Academy of Medical Sciences), Southern Medical University, Guangzhou, 510120 Guangdong People’s Republic of China; 3 Guangdong Provincial Hospital of Traditional Chinese Medicine, Guangdong Provincial Academy of Chinese Medical Sciences, and The Second Clinical College, Guangzhou University of Chinese Medicine, Guangzhou, People’s Republic of China

**Keywords:** Major depressive disorder (MDD), Suicide risk, Distress tolerance, Stressful life events, Mediating effect

## Abstract

**Background:**

Despite widespread acknowledgment of the impact of stressful life events on suicide risk, the understanding of the psychological mechanisms underlying the relationship between stressful life events and suicide risk in major depressive disorder (MDD) remain unclear. This study aim to examine whether the distress tolerance mediates the relationship between the stressful life events and suicide risk in patients with MDD.

**Methods:**

A cross-sectional study was carried out among 125 Chinese patients with MDD, mean age was 27.05 (SD=0.68) and 68.8% were females. The 17-item Hamilton Depression Rating scale (HAMD-17), the validated Chinese version of the Mini International Neuropsychiatric Interview (MINI) suicide module, Life Events Scale (LES) and Distress Tolerance Scale (DTS) were utilized to evaluate depressive symptoms, stressful life events, levels of distress tolerance, and suicide risk, respectively. Mediation analyses was used to test the mediation effect of distress tolerance on the relationship between stressful life events and suicide risk.

**Results:**

The ratio of suicide risk in patients with MDD was 75.2%. Pearson correlation analysis showed that stressful life events were positively correlated with suicide risk(*r*=0.182, *p*<0.05). Stressful life events(*r*=-0.323, *p*<0.01) and suicide risk(*r*=-0.354, *p*<0.01) were negatively correlated with distress tolerance. Mediation analyses showed that the direct path from stressful life events to suicide risk was not significant (B= 0.012, 95% confidence interval (CI) [-0.017, 0.042]). Stressful life events affected suicide risk indirectly through distress tolerance (B= 0.018, 95% CI [0.007, 0.031]), and the mediating effect accounted for 60.0% of the total effect.

**Conclusion:**

Distress tolerance completely played a mediating role between stressful life events and suicide risk. Further suicide prevention and intervention strategies should focus on increasing levels of distress tolerance in patients with MDD.

## Background

Suicide is a public health concern and the leading case of disability worldwide [1]. According to the World Health Organization (WHO) latest estimates, 703 000 people die by suicide every year globally [2]. Suicide risk is the probability of performing suicidal behavior on the basis of suicidal ideation, which is usually used to assess individual likelihood of suicide [3]. Mental disorder especially depression is one of the most important risk factors for suicide [4]. 90% of suicide victims suffer from one or more mental disorders during their lifetime, and patients with depression accounting for 59%-87% of all suicides [5, 6]. It has been found that depression is a high-risk factor for causing suicide [7]. 15 % of patients with major depressive disorder (MDD) died by suicide [8]. According to a multicenter study, Chinese patients with depressive disorder has the highest suicide rate in Asia, at 36.3% [9].

The stress-diathesis model theory of suicide considers suicidal behaviors as the result of an interaction between stress factors and individual susceptibility [10]. Suicidal behavior occurs when an individual with the diathesis is exposed to stress, which determines the behavior’s timing [10]. The individuals who experience more stressful life events are associated with increased severity of depressive symptoms, as well as increased risk for suicide [11, 12]. A strong link between stressful life events and suicide risk in patients with MDD has been consistently demonstrated that the number and severity of stressful life events have a quantity-response relationship with suicide risk [13, 14]. Although widespread acknowledgment of the impact of stressful life events on suicide risk, little is known about the underlying mechanisms that might explain effects of stressful life events on suicide risk. Few studies have sought to disentangle this relationship. Hence, exploring the psychopathological mechanism of the relationship between stressful life events and suicide risk has an important role in reducing the suicide risk in patients with MDD.

Distress tolerance is defined as trait-like individual difference variable reflecting the actual or perceived ability to experience, accept, and persist in the context of negative psychological states [15]. Moreover, distress tolerance has recently been recognized as a developmental and maintenance factor in multiple psychiatric disorder, including MDD [16]. The effects of stressful experiences on mental health outcomes were influenced by the ability of individuals to endure distress. Individuals with low levels of distress tolerance is associated with increased severity of depressive symptoms [17, 18]. Besides, distress tolerance is considered as a promising target within a variety of psychotherapeutic approaches in the context of treatments for depression [19]. Consistent with negative reinforcement models of the development of mental disorders, the lower levels of distress tolerance of individuals, the more likely to manage their emotions using some maladaptive coping strategies to avoid negative emotional status, such as substance use problems, self-harm and suicide behaviors [20–23]. Research has shown that low levels of distress tolerance are associated with increased risk for suicide [24]. In recent years, researchers have consistently documented the role of distress tolerance in suicide prevention [25, 26]. However, few studies have explored the relationship between stressful life events and distress tolerance. To our best knowledge, there are only two related studies. Such as that a study of negative life events is associated with distress tolerance in non-clinical sample of adolescence [27]. The another study has shown occupational stress would interact with distress tolerance to predict suicide risk [26]. Individuals who are exposed to stressful life events may experience negative emotions. Individuals with low levels of distress tolerance find it hard to withstand and cope with negative emotions, and are more likely to have and increased risk of suicide. However, there is no study has explored the mediation effect of distress tolerance on the relationship of the stressful life events and suicide risk in patients with MDD up to date.

Given that stressful life events were widely recognized as a risk factor of suicide, the primary aim of the current study was to examine the mediation effect of distress tolerance on relationship between the stressful life events and suicide risk in patients with MDD. Further exploring the mediating role of distress tolerance on relationship between the stressful life events and suicide risk could contribute to a better understanding of the psychological mechanisms underlying the relationship, and it is essential to develop intervention strategies in decreasing suicide risk for this susceptible population.

## Methods

### Participants

The present study was a cross-sectional study. The patients with MDD were recruited continuously from Guangdong Mental Health Center in Guangzhou, Guangdong province, China, from December 2021 to September 2022. All the patients met the following inclusion criteria: (1) patients met the criteria of the fifth edition of the Diagnostic and Statistical Manual (DSM-5) for MDD, (2) patients aged from 18 to 60 years old, (3) the score of 17-item Hamilton Depression Rating scale (HAMD-17) > 17 [28], (4) patients provided written informed consent prior to participation; Patients who met any of the following criteria were excluded: (1) patients having any other psychiatric diagnoses according to DSM-5 diagnostic criteria, (2) patients suffering from severe physical diseases, (3) patients with a history of alcohol or drug abuse in the past year, (4) patients received modified electric convulsive therapy (MECT) in the past month.

### Procedures

The investigator stated the intent and content of the study to the patients, and all the patients signed an informed consent form. Next, the trained investigator collected the sociodemographic and clinical information of the patients, then used the HAMD-17 and the validated Chinese version of the Mini International Neuropsychiatric Interview (MINI) [29] to assess the patients for depressive symptoms and suicide risk, respectively. Finally, patients completed the self-report scales.

### Demographic and clinical characteristics

Patients with MDD were required to report sociodemographic information as following: gender, age, registered residence and marital status. Additionally, clinical information was recorded, such as family history of mental illness, duration of illness for MDD and first episode.

### Assessment instruments

#### Depressive symptoms

Hamilton Depression Rating Scale 17-item (HAMD-17) was used in this study to evaluate the severity of depression in patients with MDD for the past week [28]. There are 17 items in this scale, the higher the score, the more severe of depressive symptoms. In the present study, patients with MDD with a sum score >17 on the HAMD-17 at study entry were enrolled.

#### Suicide risk

Suicide risk was assessed using the validated Chinese version of the Mini International Neuropsychiatric Interview (MINI) suicide module [29]. MINI suicide module totally include 6 questions asked by a professionally trained clinical investigator. Item 1 to item 5 respectively reflect whether there was negative thoughts, self-harm, suicide ideation, suicide plan, and suicide attempt in the latest month, and item 6 reflects whether the patients had attempted suicide in his or her lifetime. Following a previous study [30], patients were classified as without risk group (suicide risk score < 6) and suicide risk group (suicide risk score≧6). The total score ranged from 0 to 33. The higher the score, the greater the risk for suicide.

#### Stressful life events

Stressful life events were assessed using Life Events Scale (LES) which was a self-report scale contains 48 items [31]. Stimulation was divided into positive stimulation and negative stimulation according to the characteristics of the life events. The total stimulation in this study was the sum of both positive and negative stimulation.The sum score of the LES was calculated according to the following equation : total score of stressful life events = the occurrence time of life events influence × influence duration× the degree of mental impact of life events. Higher overall score of stressful life events was indicative of the higher level of stressful mental pressure the individuals perceived.

#### Distress tolerance

Simons and Gaher (2005) developed the Distress Tolerance Scale (DTS) to evaluate the (perceived) ability of individuals to experience and endure negative emotional states [32]. The DTS was consisting of 15 items and scored on a 5-point Likert scale ranging from 1 (strongly agree) to 5 (strongly disagree). This measure consists of four subscales: tolerance, appraisal, absorption and regulation. Higher score represent higher tolerance for negative emotional states. In the current study, Chinese version of DTS was utilized for assessing levels of distress tolerance [33].

### Statistical analysis

All statistics were conducted using SPSS version 26.0 (IBM Corporation, Chicago, IL). Descriptive statistics including means (M), standard deviations (SD) were generated for all continuous variables which met normal distribution and the data did not met the normal distribution were represented by median (quartile) [median (P_25_, P_75_)]. Frequencies and proportions were generated for all categorical variables. Comparison between continuous variables were performed using t-test and Mann-Whitney U t- test. Chi-square was used to test for group differences among categorical variables. Then Binary logistic regression analyse was performed for variables with significant differences between with suicide risk group and without suicide risk group in order to identify the relevant factors predicting suicide risk. Association between stressful life events, distress tolerance and suicide risk were assessed using Pearson’s correlations. *p* < 0.05 was considered to be statistically significant.

**Fig. 1:**
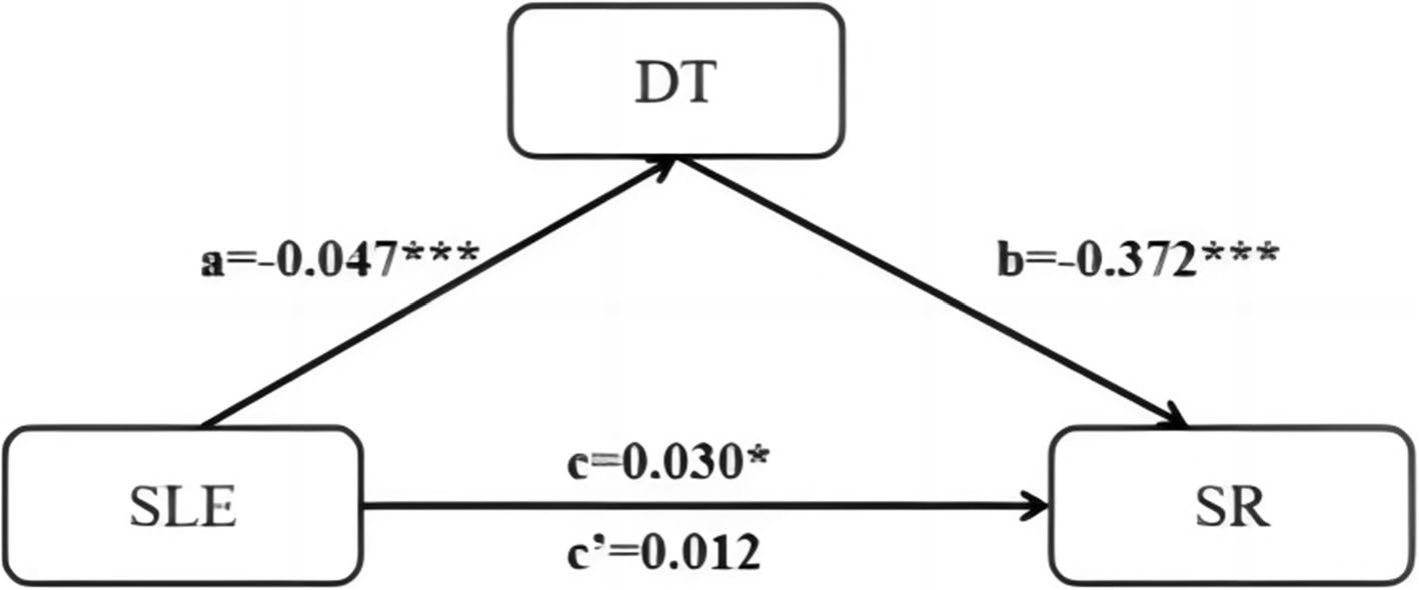
Mediation model of distress tolerance on the relationship between stress life events and suicide risk. Estimated path coefficients are refereed to a, b, c, and c’. Note: SLE: stressful life events; DT: distress tolerance; SR: suicide risk. * *p*<0.05. *** *p*<0.001.

The model 4 in the SPSS 26.0 Hayes’s PROCESS V4.1 plugin was performed for mediation effect analyses [34]. The mediation effect of distress tolerance on relationship between stress life events and suicide risk were tested (Fig. 1). Bootstrapping approach (5000 bootstrap re-samples) was used assessing the significance of the indirect effect, and the upper and lower limits of 95% confidence interval (95% CI) do not contain 0, indicating a significant mediation effect.

## Results

### Description of the sample characteristics and comparison in patients with MDD with and without suicide risk

The sample consisted of the 125 patients with MDD. There were 39 (31.2%) and 86 females (68.8%). The mean age of the study participants was years 27.50(SD=0.68). According to the scoring results of the MINI suicide module, with a cut-off value of 6, 94 (75.2%) patients with MDD were at risk of suicide. 36 (28.8%) patients with MDD had ever attempted suicide, 12 (9.6%) had attempted suicide in the last month. All of demographic characteristics (age, gender and so on) and clinical characteristics (family history of mental illness、duration of illness for MDD and first-onset) had no significant difference between suicide risk group and without suicide risk group (*p*>0.05) (Table 1). The mean scores and standard deviations (M±SD) on the HAMD-17, the LES, the DTS and the MINI suicide risk module were 22.21±4.07, 84.18±54.09, 37.67±7.91 and 10.72±8.94, respectively (Table 3). In addition, it was found that patients with MDD with suicide risk experienced more stressful life events (*p* < 0.05) and had more severe depressive symptoms (*p* > 0 .001) than patients without suicide risk. Besides, patients with MDD with suicide risk had lower levels of distress tolerance than patients without suicide risk (*p* < 0.001) (Table 1). The results of Binary logistic regression analyses showed that depressive symptoms (OR=1.478, 95% CI [1.211,1.801]) and stressful life events (OR=1.009, 95% CI [1.000,1.018]) were independent risk factors of suicide in patients with MDD. In addition, distress tolerance (OR=0.923, 95% CI [0.875, 0.975]) was independent protective factor of suicide risk in patients with MDD (Table 2).

**Table 1:** Description of the sample characteristics and comparison in patients with MDD with and without suicide risk (*n*=125)

Variables	Without Suicide risk(*n*=31)	With Suicide risk(*n*=94)	t/χ2/Z	*P*
Age (years), n (%)				
18≤age≤35	27(87.1)	77(81.9)	0.45	0.591
35<age≤60	4(12.9)	17(18.1)		
Gender, n (%)				
Male	11(35.5)	28(29.8)	0.35	0.553
Female	20(65.5)	66(70.2)		
Education (years), median (P_25_, P_75_)	15.0(13.0, 16.0)	15.0(13.0,16.0)	0.05	0.960
Marital status, n (%)				
Married	7(22.6)	25(26.6)	0.20	0.657
Single	24(77.4)	69(73.4)		
Occupation, n (%)				
Employed	4(12.9)	79(16.0)	0.17	0.781
Unemployed	27(87.1)	15(84.0)		
Residence, n (%)				
Rural	14(45.2)	38(40.4)	0.22	0.634
Urban	17(54.8)	56(59.6)		
Family history of mental illness, n (%)				
Yes	4(12.9)	19(20.2)	0.83	0.434
No	27(87.1)	75(79.8)		
First-onset, n (%)				
Yes	19(61.3)	49(52.1)	0.79	0.374
No	12(38.7)	45(47.9)		
Duration of illness for MDD (years), median (P_25_, P_75_)	1.0(0.0, 2.0)	1.0(0.0, 3.0)	0.87	0.387
Depressive symptoms, M(SD)	19.45±1.96	23.12±4.18	-6.58	0.000
Distress tolerance, M(SD)	41.39±8.50	36.45±7.35	3.12	0.002
Stress life events, M(SD)	66.71±42.09	89.95±56.51	-2.10	0.038

*M* Mean, *SD* Standard Deviation, *MDD* major depressive disorder.

**Table 2:** Binary logistic regression results for predicting suicide risk in patients with MDD (*n*=125)

Variables	B	SE	OR	95% CI	*P*
Depressive symptoms	0.390	0.101	1.478	[1.212, 1.801]	0.000
Distress tolerance	-0.080	0.028	0.923	[0.875, 0.975]	0.004
Stress life events	0.009	0.004	1.009	[1.000, 1.018]	0.041

*B* unstandardized regression coefficient, *SE* standard error, *OR* odds ratios, *CI* confidence interval

### Correlation analyses between variables

The mean scores (M) and standard deviations (SD) for each of the four variables (depressive symptoms, distress tolerance, stress life events and suicide risk) were presented in Table 3. Pearson correlation analyses revealed that suicide risk was negatively correlated with distress tolerance (*r* = -0.354, *p* < 0.01), and positively correlated with stressful life events (*r* = 0.182, *p* < 0.05) and depressive symptoms (*r* = 0.522, *p* <0.01). Besides, distress tolerance was negatively correlated with stressful life events (*r* = -0.323, *p* <0.01). Depressive symptoms were positively correlated with stressful life events (r = 0.346, *p* < 0.01) and suicide risk (*r* = 0.522, *p* < 0.01) negatively correlated distress tolerance (*r* = -0.406, *p* < 0.01) (Table 3).

**Table 3:** Correlation analyses among variables in patients with MDD (*n*=125)

Variable	1	2	3	4
Depressive symptoms	1			
Distress tolerance	-0.406**	1		
Stress life events	0.346**	-0.323**	1	
Suicide risk	0.522**	-0.354**	0.182*	1
Mean	22.21	37.67	84.18	10.72
SD	4.07	7.91	54.09	8.94

*SD* Standard Deviation

^*^p < 0.05

^**^p < 0.01

### The mediating effect of distress tolerance on the relationship between stressful life events and suicide risk

In the current study, we hypothesized that the relationship between stressful life events and suicide risk mediated by distress tolerance (Fig. 1). Stressful life events were entered as the independent variable (X), distress tolerance as mediator (M) and suicide risk as dependent variable (Y) in model 4. Results showed that stressful life events had a significant positive total effect (c) on suicide risk (B= 0.30, SE = 0.015, *p* < 0.05, 95% CI [0.001, 0.059]). The path(a) from stressful life events to distress tolerance (B= -0.047, SE = 0.012, *p* < .001, 95% CI [-0.072, -0.023]) and the path (b) from distress tolerance to suicide risk were significant (B= -0.372, SE = 0.101, *p* < 0.001, 95% CI [-0.572, -0.173]). In addition, it was found that the indirect path (a*b) from stressful life events through distress tolerance to suicide risk was significant (B= 0.018, SE=0.016, 95% CI [0.007, 0.031]). However, the direct path (c’ ) from stressful life events to suicide risk was not significant (B= 0.012, SE=0.015, *p* > 0.05, 95% CI [-0.017, 0.042]). Hence, the distress tolerance completely mediated the relationship between stressful life events and suicide risk, and the ratio of the mediating effect to the total effect was 60.0% (Table 4).

**Table 4:** Mediation analyses of distress tolerance on the relationship between stressful life events and suicide risk

Effect	Pathway	B	SE	t	p	Bootstrap 95% CI		a*b /a*b + c’(%)
						LLCI	ULCI	
	SLE→DT(a)	-0.047	0.012	-3.785	0.000	-0.072	-0.023	
	DT→SR(b)	-0.372	0.101	-3.693	0.000	-0.572	-0.173	
DE	SLE→SR(c’)	0.012	0.015	0.864	0.399	-0.017	0.042	
IE	SLE→DT→SR(a*b)	0.018	0.006	—	—	0.007	0.031	
TE	a*b + c’(c)	0.030	0.015	2.051	0.042	0.001	0.059	60.0

a*b /a*b + c’ , the ratio of mediation effect if the mediation effect (a * b) was significant and if a*b and c’ were in the same direction( both positive or both negative)

*SLE* stressful life events, *DT* distress tolerance, *DE* direct effect, *SR* suicide risk, *IE* indirect effect, *TE* total effect, *B* unstandardized regression coefficient, *SE* standard error, *CI* confidence interval, *LLCI* lower level of confidence interval, *ULCI* upper level of confidence interval.

## Discussion

The finding was that stressful life events affect suicide risk in patients with MDD indirectly through distress tolerance. This finding provided a new psychopathology perspective on the prevention and intervention of suicide risk in patients with MDD.

Overall, the ratio of suicide risk in patients with MDD was 75.2%, higher than in previous study [13]. This might due to sample selection bias that the majority of the MDD cohort were young patients. Young age was considered as one of the risk factors of suicide [35]. Compared to patients with MDD without suicide risk, patients with MDD with suicide risk had more severe depressive symptoms and more exposure to stressful life events. Patients with MDD exposed to stressful life events were more likely influenced by negative emotions which increased suicide risk. This finding was in line with previous study [13]. In addition, among patients with MDD, those with suicide risk had significantly lower levels of distress tolerance than those without suicide risk. Moreover, logistic regression analyses result showed that distress tolerance was a protective factor for suicide risk in patients with MDD. The finding of this study suggested that distress tolerance is an important indicator for the prevention and intervention of suicide in patients with MDD. Previous study also suggested that distress tolerance was considered as a feasibility indicator for suicide prevention [36].

As excepted, this study found that stressful life events were positively related with suicide risk. This finding was in line in with previous studies [13, 18]. The result showed that distress tolerance was negatively related with depressive symptoms. Lower levels of distress tolerance was associated with more severe psychological symptoms and outcomes [18]. Clinical studies have confirmed that improving distress tolerance can effectively reduce depressive symptoms, and distress tolerance has gradually become an important intervention indicator of psychotherapy [19]. Besides, it was found that distress tolerance was negatively related with stressful life events and suicide risk. Suicidal behavior has been conceptualized as a response to stress [37]. Not all individuals who have experienced stressful life events is at risk of suicide, which may be related to the individual’s own ability to endure the negative emotions when exposed to stressful life events. Patients with MDD exposed to stressful life events experienced higher levels of negative emotion, which may reduce levels of distress tolerance. Individuals who can not withstand the negative emotion are more likely to be engaged in maladaptive coping strategies (such as suicide behavior) to avoid negative emotional states [36, 38]. Lower levels of distress tolerance was associated with increased risk of suicide. This was in line with the theory of suicide cube model proposed by Shneidman that one of reasons for committing to suicide is that individuals can not withstand distress status [39].

The mediation effect analyses results showed that stressful life events can positively predict suicide risk in patients with MDD. And distress tolerance can negatively predict suicide risk in patients with MDD. This finding of distress tolerance as a predictor of suicide risk was in line with a nonclinical sample study [26]. In addition, mediation analysis revealed a significant indirect effect of stressful life events on suicide risk, and the effect was completely mediated through distress tolerance. And the ratio of the mediating effect to the total effect was 60.0%. Hence, stressful life events affect suicide risk in patients with MDD indirectly through distress tolerance. Stressful life events are uncontrollable external environmental factor. In addition, stressful life events, as the precipitants of suicide ideation and suicide attempt among patients with MDD may increase the suicide risk of MDD patients. Therefore, it is necessary to pay attention to the stressful life events experience of patients with MDD to more accurately judge the source of distress emotions , which can help to clarify the direction of suicide prevention and intervention in MDD patients. Individual distress tolerance plays an important role in mediating between stressful life events and suicide risk. Reducing the risk of suicide caused by life events by increasing the levels of distress tolerance in patients with MDD is a feasibility strategy.

This study provided a new perspective for the clinical intervention practice of suicide in patients with MDD. Clinicians should attach importance to the assessment of the levels of distress tolerance in patients with MDD, to facilitate the early identification of suicide risk and suicide prevention and intervention. Improving the levels of distress tolerance of patients with MDD can buffer the impact of stressful life events, then decrease the suicide risk. Base on the existing literature and the finding of the current study, further suicide prevention and intervention strategies should focus on increasing the levels of distress tolerance in patients with MDD.

There were several limitations to the present study. Firstly, this study was a cross-sectional study, where the stressful life events were collected retrospectively and recall bias might influence the results. Future studies should employ a longitudinal design to investigate the effect of distress tolerance on the relationship between stressful life events and suicide risk. In addition, the definition of suicide risk is not yet unified, and there are some difficulties in assessing suicide risk, and future studies should deeply explore different aspects of suicide risk, including suicide ideation and suicide attempt. Secondly, the study was conducted in a clinical sample of patients with MDD, the severity of depressive symptoms、the duration of illness for MDD and recurrent MDD may influence the levels of distress tolerance and increase suicide risk. Thirdly, the small sample size and single-center sampling limited the generalizability of the results. Finally, there were many factors that potentially affected risk of suicide in patients with MDD (e. g., the levels of social support and personality characteristics), and more other influencing factors will need to be considered in future studies.

## Conclusion

The present study found that the relationship between stressful life events and suicide risk was completely mediated through distress tolerance. The finding highlighted the importance of distress tolerance in preventing suicide risk. Clinicians should attach importance to the assessment of the levels of distress tolerance in patients with MDD. In the future, suicide prevention efforts within patients with MDD might benefit from focusing on distress tolerance improvement.

## Data Availability

Due to ethical restrictions, the present study data were not publicly available to ensure that research participants privacy is not compromised. Data for this study are available from the corresponding author, Dr. Xu.

## Abbreviations

MDD: Major depressive disorder
MINI: The Mini International Neuropsychiatric Interview
HAMD-17: Hamilton Depression Rating Scale 17-item
SLE: stressful life events
LES: Life events scale
DT: Distress tolerance
DTS: Distress tolerance scale
SR: Suicide risk
IE: Indirect effect
DE: Direct effect
TE: Total effect
B: Unstandardized regression coefficient
CI: Confidence interval
LLCI: Lower level of confidence interval
ULCI: Upper level of confidence interval
